# Kallikrein-Related Peptidase 14 Activates Zymogens of Membrane Type Matrix Metalloproteinases (MT-MMPs)—A CleavEx Based Analysis

**DOI:** 10.3390/ijms21124383

**Published:** 2020-06-19

**Authors:** Katherine Falkowski, Ewa Bielecka, Ida B. Thøgersen, Oliwia Bocheńska, Karolina Płaza, Magdalena Kalińska, Laura Sąsiadek, Małgorzata Magoch, Aleksandra Pęcak, Magdalena Wiśniewska, Natalia Gruba, Magdalena Wysocka, Anna Wojtysiak, Magdalena Brzezińska-Bodal, Kamila Sychowska, Anastasija Pejkovska, Maren Rehders, Georgina Butler, Christopher M Overall, Klaudia Brix, Grzegorz Dubin, Adam Lesner, Andrzej Kozik, Jan J. Enghild, Jan Potempa, Tomasz Kantyka

**Affiliations:** 1 Malopolska Centre of Biotechnology, Jagiellonian University, 30-387 Krakow, Poland; falkowski.katherine@gmail.com (K.F.); ewa.bielecka@uj.edu.pl (E.B.); lmsasiadek@gmail.com (L.S.); m.magoch@gmail.com (M.M.); aleksandra.pecak@gmail.com (A.P.); wisienka.magdalena@gmail.com (M.W.); grzegorz.dubin@uj.edu.pl (G.D.); 2 Faculty of Biochemistry, Biophysics and Biotechnology, Jagiellonian University, 30-387 Krakow, Poland; oliwia.bochenska@gmail.com (O.B.); plaza.karolina@gmail.com (K.P.); magda.kalinska@uj.edu.pl (M.K.); andrzej.kozik@uj.edu.pl (A.K.); jan.potempa@uj.edu.pl (J.P.); 3 Department of Molecular Biology and Genetics, Aarhus University, 8000 Aarhus, Denmark; ibt@mbg.au.dk (I.B.T.); jje@mbg.au.dk (J.J.E.); 4 Faculty of Chemistry, University of Gdansk, 80-308 Gdansk, Poland; natalia.gruba@ug.edu.pl (N.G.); magdalena.wysocka@ug.edu.pl (M.W.); anna.wojtysiak@phdstud.ug.edu.pl (A.W.); magdalena.brzezinska@phdstud.ug.edu.pl (M.B.-B.); kamila.sychowska@gmail.com (K.S.); adam.lesner@ug.edu.pl (A.L.); 5 Department of Life Sciences and Chemistry, Jacobs University Bremen, 28759 Bremen, Germany; anja.pejkovska@gmail.com (A.P.); m.rehders@jacobs-university.de (M.R.); k.brix@jacobs-university.de (K.B.); 6 Centre for Blood Research, Department of Oral Biological and Medical Sciences, University of British Columbia, Vancouver, BC V6T 1Z3, Canada; george.butler@ubc.ca (G.B.); chris.overall@ubc.ca (C.M.O.); 7 Department of Biochemistry and Molecular Biology, University of British Columbia, Vancouver, BC V6T 1Z3, Canada; 8 School of Dentistry, University of Louisville, Louisville, KY 40202, USA; 9 Broegelmann Research Laboratory, Department of Clinical Science, University of Bergen, 5020 Bergen, Norway

**Keywords:** kallikrein 14, membrane-type MMP, CleavEx, fusion protein, zymogen activation

## Abstract

Kallikrein-related peptidases (KLKs) and matrix metalloproteinases (MMPs) are secretory proteinases known to proteolytically process components of the extracellular matrix, modulating the pericellular environment in physiology and in pathologies. The interconnection between these families remains elusive. To assess the cross-activation of these families, we developed a peptide, fusion protein-based exposition system (**Cleav**age of **ex**posed amino acid sequences, CleavEx) aiming at investigating the potential of KLK14 to recognize and hydrolyze proMMP sequences. Initial assessment identified ten MMP activation domain sequences which were validated by Edman degradation. The analysis revealed that membrane-type MMPs (MT-MMPs) are targeted by KLK14 for activation. Correspondingly, proMMP14-17 were investigated in vitro and found to be effectively processed by KLK14. Again, the expected neo-N-termini of the activated MT-MMPs was confirmed by Edman degradation. The effectiveness of proMMP activation was analyzed by gelatin zymography, confirming the release of fully active, mature MT-MMPs upon KLK14 treatment. Lastly, MMP14 was shown to be processed on the cell surface by KLK14 using murine fibroblasts overexpressing human MMP14. Herein, we propose KLK14-mediated selective activation of cell-membrane located MT-MMPs as an additional layer of their regulation. As both, KLKs and MT-MMPs, are implicated in cancer, their cross-activation may constitute an important factor in tumor progression and metastasis.

## 1. Introduction

The extracellular matrix (ECM) consists of a complex array of locally secreted macromolecules interacting as a meshwork of proteins, glycoproteins, glycosaminoglycans, and proteoglycans [1]. Proteases present within the ECM are essential for turnover of structural proteins or core protein components. In addition, proteolytic enzymes are required for the activation of growth factors and other ligands of cell surface receptors. Thereby, proteolytic processing of ECM components and the molecules stored therein provides both the structural organization and essential initial steps in cell-cell communication. By initiation of the signal transduction, proteases effectively regulate gene transcription eventually impacting on cell differentiation, proliferation, growth, and apoptotic programs [2]. Proteolytic enzymes essential for ECM homeostasis predominantly belong to the families of a disintegrin and metalloproteinase (ADAMs) [3], matrix metalloproteinases (MMPs) [1], and tissue kallikrein-related peptidases (KLKs) [4]. The MMP family is considered especially important for ECM remodeling [5,6]. In particular, the MMP proteases of the membrane-type subfamily (MT-MMPs) are essential in regulating cell migration during physiological wound healing [7] but are also implicated in cancer progression as they often involve in the initial steps of cancer cell metastasis [8,9,10]. Specifically, MMP14 (MT1-MMP) associates with integrins in endothelial cells at distinct cellular domains, which may initiate cell junction processing during invasion [11]. Furthermore, MMP14 is attributed as one of the main MT-MMPs implemented in activating cancer associated proMMP2 [12].

It is corroborated that the activation of MT-MMPs happens intracellularly by means of furin-mediated processing such that the active forms of MT-MMPs decorate the cell surface right upon secretory release of cellular products by exocytosis. This pathway is not exclusive, as extracellular processing of proMT1-MMP (proMMP14) has been reported [13,14]. About one-third of all proMMPs activation domains contain an Arg/Lys-rich motif, compatible with furin specificity, but also targeted by serine proteases, particularly plasmin [15,16] and transmembrane serine proteinases hepsin and TMPRSS2 [17].

The family of human kallikrein-related peptidases consists of a total of 15 different serine proteases with trypsin-like, chymotrypsin-like, or mixed substrate specificity. Physiological roles of KLKs include regulation of cell growth and tissue remodeling [18]. Typically, KLKs and MMPs are co-expressed in many cell types and tissues [19,20,21,22]; therefore, understanding their interactions may provide a novel perspective for deciphering regulation of MMP activities in health and disease. A recent report has identified a pericellular network of KLK4, KLK14, MMP3, and MMP9 that are involved in initiation and promotion of prostate cancer cell metastasis [17]; however, the function of KLK14 in this complex was not identified.

Herein, we employed a new unbiased technique CleavEx (**Cleav**age of **ex**posed amino acid sequences), which allowed us to test for the activation of MMP zymogen forms by KLK14. Our approach was based on the recombinant attachment of an MMP activation site sequence to the proteolytically stable heme-binding (HmuY) protein. Hybrid proteins displaying all 23 activation sequences of human proMMPs was expressed and purified. These proteins were then incubated with KLK14 and the generated products were subsequently analyzed to identify the potential processing. CleavEx analysis was followed by investigation of KLK14-mediated processing of native proMMP14-17. Lastly, we identified the cell surface processing of proMMP14 by KLK14. To our knowledge, this is the first study examining the potential of KLK family member to activate the proMMP family.

## 2. Results

### 2.1. CleavEx_proMMP_ Fusion Protein Screening

**Figure 1:**
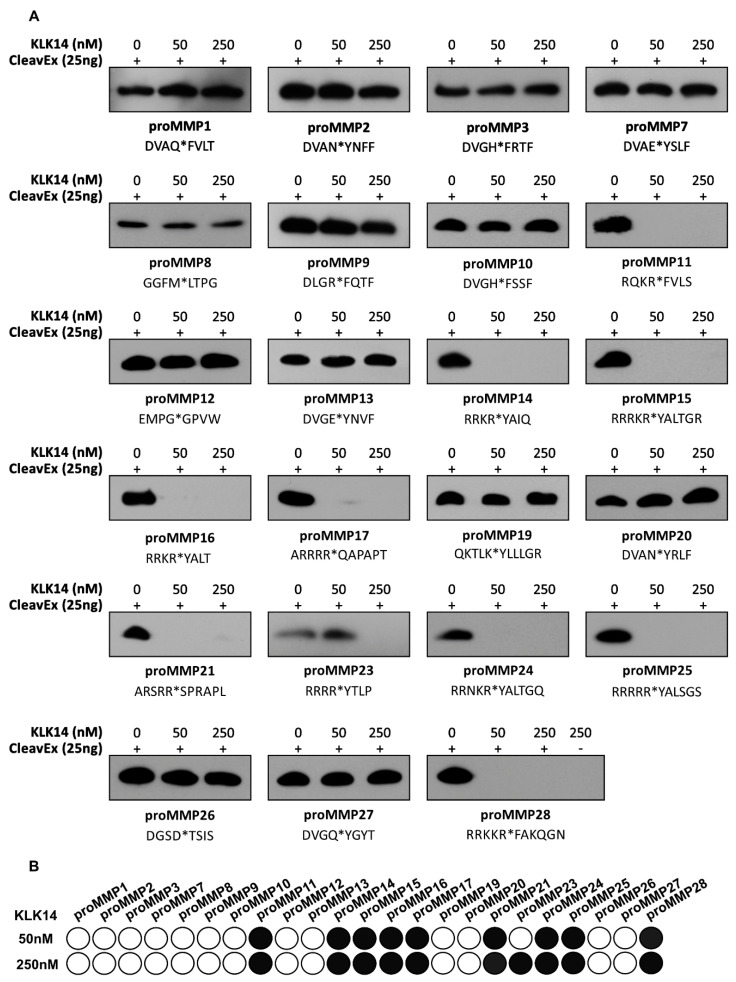
Effect of KLK14 on the CleavEx_proMMP_ fusion proteins. (**A**) Western blot analysis of each 25 ng CleavEx_proMMP_ protein incubated with 50 and 250 nM KLK14 after 1 h at 37 °C. Each fusion protein with its respective activation sequence is listed with the native site of hydrolysis indicated by an asterisk. (**B**) Schematic representation of the CleavEx_proMMP_ fusion proteins from the Western blots in panel A. Scoring was performed by densitometry analysis using ImageJ. The shading is based on the quartile of change: 100–75% of control sample intensity is presented as white (no degradation); 75–50% as light grey; 50–25% as dark grey; and 25% and lower as black. KLK = kallikrein-related peptidase; CleavEx = **Cleav**age of **ex**posed amino acid sequences; MMP = matrix metalloproteinase.

Hybrid proteins representative of each individual MMP propeptide sequence were created such that they are covalently engineered onto the proteolytic-resistant bacterial protein HmuY [23]. The hybrid proteins additionally featured an attached N-terminal extension constituting a HisTag followed by the respective proMMP activation sequence. This resulted in generating a total of 23 individual proteins, each exposing a sequence corresponding to the activation domain of the respective human prometalloproteinase. To verify the effectiveness of KLK14 in recognizing these peptide sequences, 25 ng of each hybrid protein was incubated with increasing concentrations of KLK14 (50 and 250 nM; 1:1000 and 1:200 KLK14:CleavEx_proMMP_ molar ratio). The results were visualized by a HisTag-specific Western blot, where the loss of signal was indicative of the release of the HisTag via proteolytic removal of the fragment (Figure 1A). The stability of the full-length HmuY protein was verified independently, and no KLK14-mediated degradation was observed (data not shown). Among all 23 hybrid proteins tested in this manner, the sequences corresponding to proMMP11, proMMP14-17, proMMP21, proMMP24-25, and proMMP28 were recognized with highest effectiveness. This means that KLK14 at a concentration of 50 nM was able to completely remove the HisTag from the respective hybrid proteins with HmuY. Note that CleavEx_proMMP23_ was not cleaved at 50 nM KLK14 but completely removed when 250 nM KLK14 was used.

To visualize the efficiency of cleavage within the sequence motives of different CleavEx_proMMPs_ by KLK14, the Western blot results were scored and presented as a grey scale map (Figure 1B). In addition, all hydrolyzed CleavEx_proMMPs_ at 50 nM KLK14 were further analyzed at 5, 25, and 50 nM KLK14 (Figure S1). The data indicated a striking selectivity of KLK14 towards particular proMMP sequences. To determine which peptide bonds were hydrolyzed within the activation domains, and to exclude the possibility of HmuY cleavage by KLK14, N-terminal sequencing of each hydrolyzed product of the CleavEx_proMMP_ reactions was performed. The determined N-termini nearly uniformly confirmed the expected P1 recognition site for KLK14 (Table 1). All of the CleavEx hybrid proteins, except for CleavEx_proMMP21_, were hydrolyzed at the activation-specific location expected for KLK14.

### 2.2. Processing of Recombinant proMMPs by KLK14

**Figure 2:**
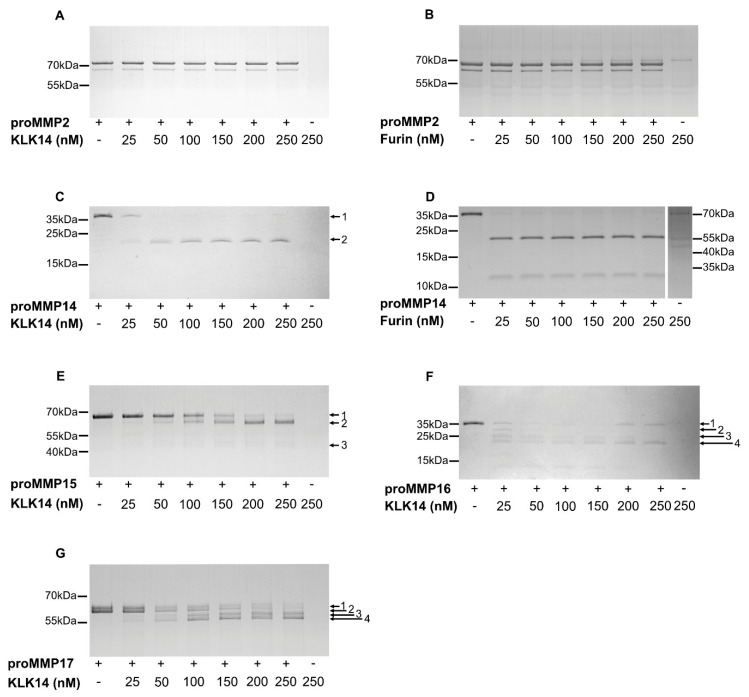
Concentration-dependent processing of proMMPs by KLK14. Each respective proMMP was incubated with increasing concentrations of KLK14 (**A**,**C**,**E**–**G**) or furin (**B**,**D**) for 1 h at 37 °C in the presence of 5 µM batimastat, and the reaction products were analyzed by SDS-PAGE and visualized with Coomassie staining. Bands denoted with arrows were identified by N-terminal sequencing using Edman degradation (Table 2). KLK = kallikrein-related peptidase; MMP = matrix metalloproteinase.

Scanning of the hybrid proteins allowed for the selection of proMMPs that are likely activated by KLK14 in situ. To verify this contention, commercially available proMMP14, proMMP15, proMMP16 and proMMP17 were analyzed for KLK14-mediated activation in vitro. Firstly, we investigated the concentration requirements of KLK14-dependent hydrolysis of each proMMP. To this end, each proform was incubated with increasing concentrations of KLK14 for 1 h (approximately from 1:65 to 1:5 KLK14:MMP molar ratio) and was then subjected to SDS-PAGE analysis. During incubation, the pan-specific MMP inhibitor batimastat was present in the reaction mixtures to prevent autoactivation of the proMMPs. ProMMP2 which was unaffected by KLK14 in the CleavEx system served as the negative control (Figure 2A). In parallel, furin was also analyzed with both, proMMP2 and proMMP14 as a negative and positive control, respectively (Figure 2B,D). All four proMMPs tested were effectively cleaved in the presence of KLK14, even at the highest substrate:enzyme molar ratios with discrete bands at molecular masses corresponding to the loss of the ~10 kDa propeptides. Moreover, the extent of proteolysis progressed in a concentration-dependent manner with 50 nM KLK14 (approximately 1:32 KLK14:MMP14 molar ratio) being sufficient to completely process proMMP14 (Figure 2C). Interestingly, in contrast to furin-mediated activation of MMP14, KLK14 incubation did not result in the accumulation of the ~12 kDa prodomain (Figure 2C,D). Higher concentrations of KLK14 were required for the full processing of other analyzed proMMPs. Nonetheless, 100 nM KLK14 was sufficient to process proMMP15-17 (approximately 1:16 KLK14:MMP15-17 molar ratio) and the hydrolyzed products remained stable up to 250 nM KLK14 (Figure 2E–G). Collectively, these results indicate that KLK14-mediated proMMP processing released stable, processed forms of MMP14-17.

In addition to the products with the expected 10 kDa shift, several other transient forms were observed and subjected to Edman sequencing for unambiguous identification. The N-terminal analysis allowed for the assignment of the cleavage sites within the proform sequence (Table 2). First, the N-termini of all of the intact proforms, denoted as band 1, were confirmed as corresponding with the reported sequences of proMMPs, providing an internal quality control. The single product, band 2_MMP14_, generated by KLK14-mediated proMMP14 processing, corresponded to the expected RRKR^111^↓YAIQ sequence, previously identified as the activation site [13]. Similarly, KLK14-mediated proMMP15 hydrolysis resulted in the accumulation of band 2_MMP15_, *PG*KR^131^↓YALT, consistent with the expected N-terminal sequence of the mature MMP15 [24]. Of note, despite the amino acids 128 and 129 being modified by the manufacturer from native arginine and arginine to proline and glycine, as indicated by italics above, KLK14 was able to generate the expected MMP15 active form. Furthermore, an additional product denoted as band 3_MMP15_ was detected to be DLRG^298^↓IQQL. Surprisingly, this product did not correspond to the expected KLK14 preference and rather was consistent with MMP15 specificity [25]. ProMMP16 processing lead to the formation of the KLK14-produced mature form, as band 4_MMP16_ corresponded to the anticipated RRKR^119^↓YALT hydrolysis. N-terminally truncated proforms were detected as band 2_MMP16_ and 3_MMP16_; however, only the N-terminal of band 3_MMP16_ (KKPR^100^↓CG) corresponded to the expected KLK14 specificity, while band 2_MMP16_ (ALAA^75^↓MQ) was consistent with MMP16 specificity [25]. ProMMP17 processing resulted in the generation of 4 bands. The first two bands, 1_MMP17_ and 2_MMP17_, observed in the untreated sample, were detected with identical N-termini A^39^PAPA, indicating the presence of differences in the C-terminal region of these proforms. KLK14 was able to process both proforms which resulted in the same sequential motif of TQAR^122^↓RRPQ (bands 3_MMP17_ and 4_MMP17_), three residues upstream of the expected native activation site.

**Figure 3:**
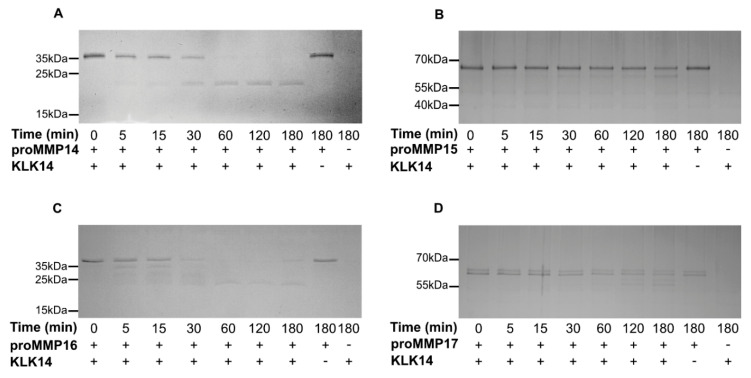
Recombinant human proMMP14,15, 16, and 17 are processed by KLK14 in a time dependent manner. (**A**–**D**) Respective proMMPs were incubated with KLK14 for 5, 15, 30, 60, 120, and 180 min, at 37 °C in the presence of 5 µM batimastat. The reaction products at indicated timepoints were analyzed by SDS-PAGE and visualized via Coomassie staining. KLK = kallikrein-related peptidase; MMP = matrix metalloproteinase.

To follow up on the concentration analysis, a time-course experiment was employed to detect product accumulation by SDS-PAGE. Specific time points were analyzed for 3 h with either 50 nM KLK14 (proMMP14) or 100 nM KLK14 (proMMP15, proMMP16, and proMMP17) to observe the processing of each respective proMMP (Figure 3). Within 5 min, the stable product, with the molecular weight corresponding to the active MMP14 form, began to appear and near complete processing was observed after 3 h, resulting in the accumulation of the mature MMP14 form (Figure 3A). In contrast, MMP15 degradation progressed more slowly with the band corresponding to the processed active form appearing vaguely after 30 min. Again, the product accumulated as a stable band, yet proMMP15 was not fully processed, even after 3 (Figure 3B). On the other hand, proMMP16 products accumulated already within 5 min. All four bands identified by N-terminal sequencing appeared early, illustrating the efficient processing by KLK14. The fully matured active form appeared as the main band after 1 h of reaction and remained stable for the whole 3 h of incubation (Figure 3C). Again, similarly to proMMP15, proMMP17 maturation was not as efficient, as lower-molecular weight bands started to appear after 2 h of reaction, and complete processing of the proform could not be observed during the incubation (Figure 3D).

### 2.3. Functional Activation of proMMPs by KLK14

**Figure 4:**
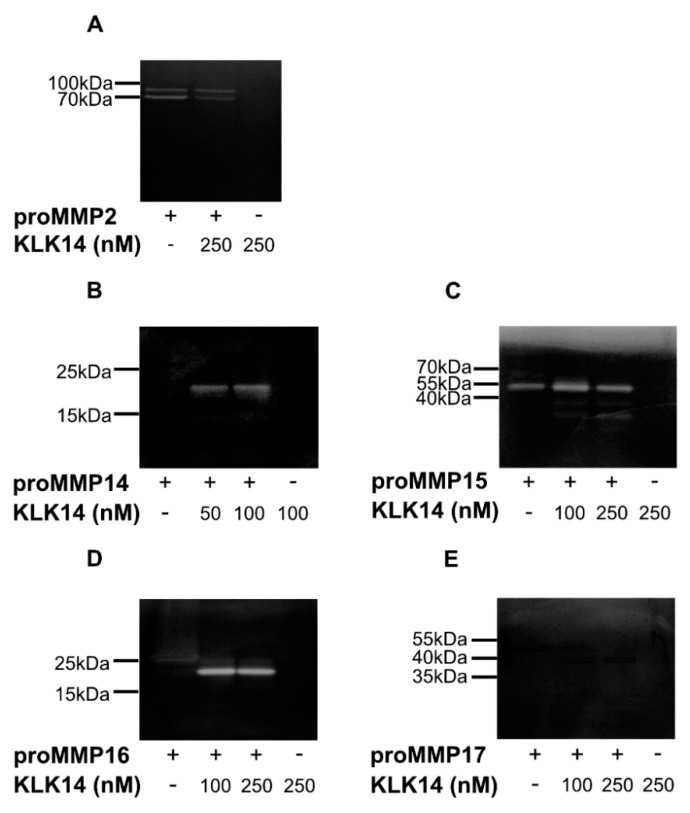
Gelatin zymography of proMMPs by KLK14-mediated processing. Activation of proMMPs by KLK14 results in a fully functional mature enzyme. Each proMMP was incubated with the indicated concentrations of KLK14 for 1 h at 37 °C. The reaction was stopped by the addition of KLK14-specific inhibitors, and the reaction mixture was analyzed by SDS-PAGE, followed by a zymogram with gelatin as a substrate. The proMMP2 (**A**) negative control was not activated. ProMMP14 (**B**), proMMP15 (**C**), and proMMP16 (**D**) were activated, whereas proMMP17 (**E**) did not show hydrolysis of gelatin; yet a shift corresponding to the loss of the profragment was observed (note that an amino acid substitution was introduced in proMMP17 by the manufacturer (R&D Systems, Abingdon, United Kingdom)). KLK = kallikrein-related peptidase; MMP = matrix metalloproteinase.

Each analyzed proMMP was activated with 50, 100, or 250 nM KLK14 for 1 h at 37 °C followed by a gelatin zymography. No KLK14 activity was detected under experimental conditions, as evident by the lack of clear bands in the KLK14 control lanes (Figure 4A–E). As previously mentioned, proMMP2 was used as a negative control. Regardless of proMMP2 alone displaying residual activity on the zymogram gel, KLK14 treatment resulted in no observable processing after 250 nM KLK14 incubation (Figure 4A). In contrast, gelatinolytic activity and the expected molecular mass shifts were observed for proMMP14–16. At both 50 and 100 nM KLK14, proMMP14 was processed to its active form with no difference in band intensity at either concentration (Figure 4B), indicating KLK14 concentrations equal or below 50 nM as sufficiently efficient convertase. Interestingly, a portion of active MMP15 was observed with no treatment of KLK14, which suggested self-activation during the 1-h incubation (Figure 4C). Furthermore, KLK14 treatment of proMMP15 resulted in enhanced activation and active band accumulation at the expected molecular weight of around 55 kDa at 100 nM KLK14, in which intensity slightly decreased at 250 nM KLK14. In addition, lower molecular mass bands (~35 kDa) were observed at both KLK14 concentrations, also displaying gelatinolytic activity. Part of these fragments likely corresponded to the DLRG^298^↓IQQL truncation identified from N-terminal sequencing, as well as MMP15 self-degraded products, since no MMP inhibitor is present in the activity assay. As expected, proMMP16 was activated, and its gelatinolytic activity was stable at both KLK14 concentrations (Figure 4D). Lastly, there was no observable proMMP17 gelatinolytic activity, despite the expected molecular mass shift of its mature form, visible in the zymogram gel (Figure 4E). MMP17 is unlikely acting as a gelatinase, which is consistent with some previous reports of others [26].

### 2.4. Comparison of Functional Activation of proMMP14 by KLK14 and Furin

**Figure 5:**
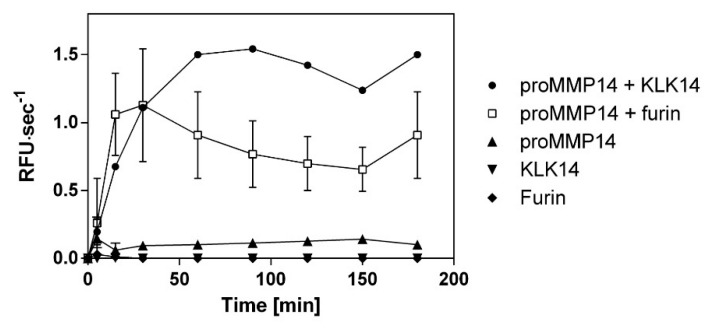
Comparison of recombinant human proMMP14 activation by KLK14 and furin as a functional peptidase. ProMMP14 (10 nM) was incubated with KLK14 (3 nM) and furin (3 nM) in the presence of a fluorogenic substrate Mca-KPLGL-Dpa-AR-NH2. The final concentration of the substrate was 10 μM in the MMP reaction buffer (50 mM Tris, 3 mM CaCl2, 1 µM ZnCl2, pH 8.5). Hydrolysis was recorded for 180 min at 37 °C. The velocities were plotted in triplicates as mean ± SD using GraphPad Prism. Please note that the error bars may be occluded by the point markers and are not visible for some of the data points. KLK = kallikrein-related peptidase; MMP = matrix metalloproteinase.

Native recombinant proteins illustrated that furin and KLK14 have similar proMMP14 activation efficiency. Therefore, to confirm the equivalence of KLK14 and furin processing, increase in proMMP14 activity on a synthetic substrate was observed in parallel upon treatment with these convertases. Observed activity of KLK14/furin-treated MMP14 increased evenly for 30 min. However, after 1 h, the KLK14-mediated activation lead to 2-fold higher MMP14 activity when compared to furin. This difference remained consistent for the remaining 180 min of hydrolysis. As expected, neither KLK14 or furin had no observable activity on the MMP14 fluorescent substrate (Figure 5).

### 2.5. Cell Surface Processing of proMMP14 by KLK14

**Figure 6:**
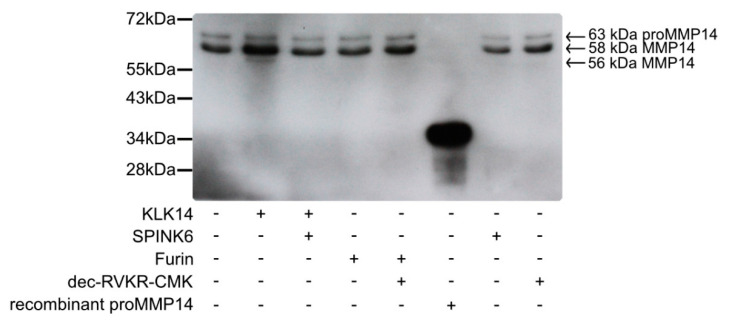
Processing of cell surface proMMP14 by KLK14. Murine fibroblasts stably expressing human MMP14 (MT1-MMP) were treated with KLK14 and furin. Selective inhibitors serine protease inhibitor Kazal-type 6 (SPINK6) (KLK14) and dec-RVKR-CMK (furin) were used to inhibit KLK and furin in the control samples. Cell surface proteins were then biotinylated, and streptavidin bead immunoprecipitates were subjected to immunoblotting using an anti-MMP14 antibody. Each sample contained the 63 kDa proMMP14 form, whereas an increase in the active 58 kDa MMP14 form was observed after KLK14 incubation. Additionally, a lower molecular weight MMP14 form at 56 kDa was detected only in the KLK14 treated sample. KLK = kallikrein-related peptidase; MMP = matrix metalloproteinase.

A key step in demonstrating whether KLK14 activates proMMP14 at the cell surface is to validate whether it can occur in a cellular system. For this, murine fibroblasts stably expressed with human MMP14 (MT1-MMP) were grown to obtain a cell density of 1 million cells/well. Cells were treated with active KLK14 and furin, along with appropriate controls, and surface protein biotinylation was performed, followed by immunoprecipitation of the biotinylated proteins and a MMP14-specific Western blot. The specific bands for MMP14 were seen with a corresponding ~63 kDa and ~58 kDa molecular mass (Figure 6) and were interpreted as proMMP14 and active MMP14, respectively, in accordance with the previous records [27,28]. Upon cell stimulation with 50 nM KLK14 for 30 min, an increase in intensity of the 58 kDa band was observed. Furthermore, a processed MMP14 form was detected at a slightly lower molecular mass of ~56 kDa. This form also appeared in a KLK14 concentration-dependent manner and was stable up to 500 nM KLK14 (Figure S2). Interestingly, furin did not facilitate an increase in intensity in any of the bands, nor did it produce any lower-molecular weight products, detectable on Western blot.

## 3. Discussion

Matrix metalloproteinases are essential enzymes in the maintenance of tissue homeostasis, namely by regulation of its self-reorganization, through growth factor activation and processing, as well as involvement in the regulation of immune cell functions [29]. Herein, we present biochemical evidence of selective activation of membrane-type MMPs by the kallikrein-related protease family member KLK14.

Our assessment is based on an unbiased approach, where all proMMP-specific activation sequences were displayed utilizing a hybrid protein system, allowing for easy production, purification and fragmentation analysis. The described herein CleavEx (**Cleav**age of **ex**posed amino acid sequences) system facilitated a rapid selection of KLK14-specific candidate proMMPs for further analysis with recombinant proteins. The carrier protein, HmuY, is produced by the bacterium responsible for periodontitis, *Porphyromonas gingivalis*, which uses an arsenal of proteolytic enzymes as virulence factors [30]. Stability studies indicate an unsurpassed resistance of the HmuY protein to proteolytic digestion by trypsin and bacterial proteinases alike [23] and suggest enhanced stability in situ (i.e., in inflamed tissue). Furthermore, structural studies revealed that the N- and C-termini of HmuY are exposed to the solvent and are thus easily accessible, while the overall globular structure of HmuY ensures good solubility and ease of folding when produced recombinantly [23]. The N-terminal HisTag serves as an affinity label for purification and additionally as a degradation-specific tag for follow-up of peptide bond hydrolysis, even in complex protein mixtures. Previously, a similar approach, but using a modified fibroblast growth factor-1 (FGF-1), was implemented by another group in the analysis of a proKLK cascade activation [31,32] and MMP-dependent activation of KLKs [33]. Yet, we believe the choice of HmuY used herein to be superior. The approximate 25 kDa molecular masses of the CleavEx fusion proteins and the ~2.5 kDa N-terminal fragment removal allowed for an easy follow-up of the reaction products by SDS-PAGE and Western blot in comparison to the FGF-1-based system, characterized by a ~1kDa shift only.

**Figure 7:**
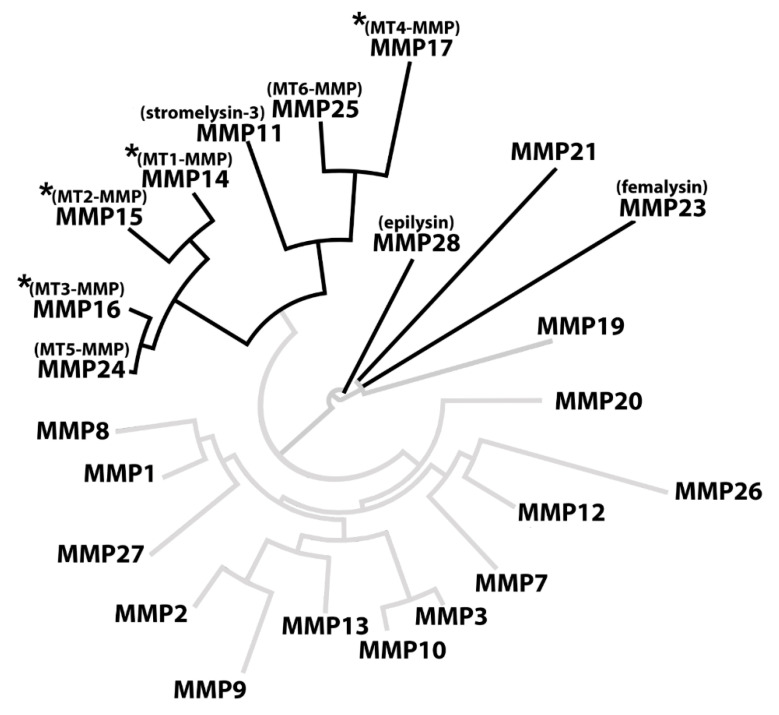
KLK14-activated MMPs cluster together in phylogenetic analysis. The full sequences of all human MMPs were obtained from the uniport database and analyzed in BioEdit using built-in multiple alignment ClustalW algorithm. The resulting alignment was visualized using an online tool (www.phylogeny.fr). The full black branches represent the MMPs in which the profragment activation sequence was hydrolyzed by KLK14. An asterisk denotes MMPs processed by KLK14 as verified in vitro. MMP = matrix metalloproteinase; MT-MMP = membrane-type matrix metalloproteinase.

Out of all 23 sequences, a total of 10 CleavEx_proMMP_ fusion proteins were hydrolyzed by KLK14, specifically the ones corresponding to membrane-type MMPs (MMP14-17 and MMP24-25), stromelysin-3 (MMP11), MMP21, femalysin (MMP23), and epilysin (MMP28) activation sequences (Figure 1). Importantly, CleavEx_proMMP_ hydrolysis by KLK14 was specific and limited to sequences derived from a subset of metalloprotease zymogens. Strikingly, the majority of all proMMPs recognized by KLK14 in the CleavEx analysis, cluster together constituting the group of membrane-type (MT) MMPs. This subgroup of the MMP proteinases is bound to the plasma membrane, either by type I transmembrane domains (MMP14-16, MMP24) or by means of phosphatidylinositol-anchors attached to the protein chains (MMP17 and MMP25) [20]. Therefore, recognition by KLK14 reflects not only the similarities in the activation domain of MT-MMPs but also the common evolutionary ancestry and functional homology of the processed proteinases. Together, these results indicate that the group of cell-surface proMMPs can be nearly exclusively targeted by KLK14-mediated activation (Figure 7).

Commercially available recombinant proMMPs were investigated to confirm the initial CleavEx_proMMP_ analysis. KLK14 was an effective convertase for proMMP14 and proMMP16 but less-efficiently, for proMMP15 and proMMP17 in a time dependent manner (Figure 3). N-terminal sequencing of the concentration-dependent KLK14-released products identified the expected hydrolysis sites for proMMP14, proMMP15, and proMMP16, respectively, indicating native-like processing of these enzymes by KLK14 [13,24] (Table 2). ProMMP14 processing by KLK14 and furin revealed a single band corresponding to the active form (Figure 2C,D). No ~12 kDa prodomain was visible upon KLK14 treatment, while furin released an intact profragment observed on SDS-PAGE. This KLK14-mediated prodomain processing may be vital in the disruption of the non-covalent association between the MMP14 prodomain and catalytic domain. As observed in the activity assay using a synthetic substrate, KLK14-mediated MMP14 activation was more efficient than furin, which may be due to the prodomain still being non-covalently attached serving as inhibitor of MMP14 [34] (Figure 5). In addition, the identified G^298^–I^299^ internal cleavage of MMP15 was located in the hinge region connecting the catalytic domain and hemopexin domain [35] (Table 2). It is difficult to predict the impact of this cleavage on the protease activity of the resulting MMP15 and its specificity towards protein substrates. However, the appearance of a corresponding band in the zymogram analysis indicated ability to cleave gelatin and suggested that this specific auto-processing by initial KLK14-mediation may be responsible for shedding of the active catalytic domain of MMP15 from the cell surface (Figure 4C). Interestingly, the cell surface localization of MMP15 was essential for its ability to cleave collagen in cell-based assays, and it was required for exhibition of invasive cancer cell phenotypes [36,37], while soluble MMP15 was found to display a nearly 13-fold higher activity on triple-helical substrates compared to the cell-surface located form [38]. This suggests, that release of the mature MMP15 from the cell surface may be an important regulatory step, resulting in enhanced triple helical collagen I degradation. Furthermore, lower concentrations of KLK14 induced step-wise MMMP16 profragment processing, as revealed by two truncated proforms, one form corresponding to KLK14 specificity (KKPR^100^↓CG) and the other to the specificity of MMP16 [25] (Figure 2F and Table 2). The processing of KKPR^100^↓CG, illustrates that KLK14 recognizes the site adjacent to cysteine-sulfhydryl group within the prodomain that associates with the catalytic zinc, which may disrupt the noncovalent interaction vital in releasing an active MMP16. Lastly, two distinct bands were present in the proMMP17 untreated samples both with identical N-termini, suggesting the difference between these products is in the C-terminal region (Figure 2G). KLK14 processed both proMMP17 forms and generated two bands, both again with the same N-termini, three amino acids upstream at the motif TQAR^122^↓RRPQ (Table 2). However, as described in the manufacturer documentation, the proMMP17 activation site R^125^ was exchanged to P^125^ therefore the native activation site is not accessible for proteolysis, which in turn targets a secondary activation site.

The verification of the hydrolysis sites at the expected activation location (except for proMMP17) indicates the proper processing of the proMMP by KLK14; however, it does not confirm the production of the active metalloproteinase from its zymogen form. To validate that KLK14-mediated processing does in fact release mature and functional MMP proteinases, gelatin zymography was employed (Figure 4). It confirmed MMP14, 15, and 16 as functional proteinases since clear bands appeared at the expected molecular mass shifts of processed MMPs. Despite the correct mass shift, proMMP17 displayed no gelatinolytic activity, either due to the abovementioned modification of the native activation site or the natural low activity of MMP17 on gelatin as a substrate [26]. It is worth noting, however, that all commercially available proMMP14-17 preparations do not contain the membrane-interacting domain, located in the C-terminal region of the mature forms, which may impact activation kinetics in vivo. Therefore, demonstrating whether KLK14 activates MT-MMP in a cellular system provides increasing evidence of an inter-activating KLK and MT-MMP network.

Recently, a KLK4/KLK14 activity system on the cell surface of COS-7 cells was described [17]. The authors propose a cell surface-located organization of a proteinase complex, containing hepsin and transmembrane serine protease 2 (TMPRSS2), two membrane-bound serine proteinases, which recruit KLK4 and KLK14 and activate proMMP3 and proMMP9. In this complex, KLK14 undergoes specific activation and accumulates in the active form, bound to the plasma membrane by a so far unknown mechanism. The role of cell surface-bound KLK14 in that system was not identified. However, based on the data presented in our study, we believe and propose that the proximity of MT-MMPs to membrane-attached, proteolytically active KLK14 may provide a yet undescribed platform for the activation of MT-MMPs.

As exemplified by the archetypical member of the subfamily, MT1-MMP (MMP14), this subclass of MMPs was reported to undergo furin-dependent activation in the compartments of the late secretory pathway; however, a body of research challenges this earlier assumption [13]. An alternative activation pathway was identified in furin-deficient RPE.40 cells which were able to display active MMP14 with the same N-terminus as the native form found in furin-expressing COS-7 cells [13]. As furin expressing CHO-K1 cells processed the membrane-bound form to the same extent as RPE.40 cells, while also converting the soluble form of MMP14, the authors concluded, that furin is required only for the maturation of the soluble form. Other, yet unidentified enzymes are responsible for the processing of membrane bound MMP14 [13]. Similarly, the non-constitutive, furin-independent processing of proMMP14 was also reported to occur in fibrosarcoma HT-1080 and CCL-137 normal fibroblast cells, where the 63 kDa proform of MMP14 was detected in both, the tumor-derived cell line and in normal cells upon PMA stimulation [39,40]. Thus, a furin-independent activation pathway needs to be further elucidated since active MMP14 was still present in furin-deficient cells [41,42,43].

MMP14 is expressed quite low in cells normally between 100,000–200,000 sites/cell, which makes it difficult to perform biochemical analysis and investigate functional parameters [44]. To delve into its processing, the *MT1-MMP* (*MMP14*) gene is transfected either transiently or stably, which results in MMP14 overexpression, leading to a plethora of cell surface forms; 63 kDa proenzyme, 54 kDa enzyme; and a 39 – 45 kDa degradation products [27]. The work presented here shows MMP14 to be processed on the cell surface by KLK14 using murine fibroblasts stably overexpressing MMP14 (Figure 6). The ~63 and 58 kDa forms detected by immunoprecipitation are consistent with previous reports and were identified as proMMP14 and active MMP14, respectively [27,28]. Indeed, the intensity of the 58 kDa band increased upon KLK14 treatment. In addition, a lower-molecular weight MMP14 form of around 56 kDa appeared. Intriguingly, this may present an alternative location of processing the membrane anchored proMMP14 by KLK14, as potential locations corresponding with KLK14 specificity are present ~1 and 4 kDa downstream in the MMP14 sequence (TPK^134^ and IRK^146^, respectively). Furthermore, in our experiments, furin did not process cell surface proMMP14, also consistent with previous reports, indicating that furin acts exclusively in the Golgi apparatus and/or on soluble, not-membrane-bound forms of MMP14 [41].

Researchers confirmed that there is a 2-step activation process for MT1-MMP. The linkage (RRPRC^93^GVPD) in the prodomain maintains the latent proenzyme by chelating the cysteine-sulfhydryl group to the active site zinc, which furin hydrolysis alone is not enough to disrupt the interaction [34]. Most importantly, mutant forms of the MMP dependent cleavage site (PGD↓L^50^) and furin activation site (RRKR^111^↓Y^112^) sequences in HT 1080 cells showed both proenzyme and enzyme forms of MMP14 on their cell surface, thus again confirming a furin-independent pathway of MMP14 processing [34]. This cell-surface processing sheds more light on the potential of various secreted MMP14 forms to be differentially regulated in normal and cancer cells by extracellular KLK14. In addition to KLK14 hydrolyzing the furin recognition site (RRKR^111^↓Y^112^), KLK14 may also recognize the trypsin-like sequence, RRPR^92^CGVPD, which is specifically adjacent to the site of the cysteine-sulfhydryl group within the prodomain that associates with the catalytic zinc in the active site. In the work presented here (Figure 2C,D), when recombinant proMMP14 was incubated with furin, the released prodomain was intact and visible, which confirms furin hydrolysis is limited to only the furin recognition site [45]. On the other hand, KLK14 further degraded the released prodomain since it was no longer visible at 12 kDa as compared to furin. Thus, this indicates the additional KLK14 hydrolysis in the prodomain, which may be vital in the release non-covalent association of the prodomain and catalytic domain once KLK14 or furin cleave at the furin recognition site. Intriguingly, KLK14 recognized this highly conserved cysteine linkage sequence, KKPR^100^↓CGVPD, within the prodomain of recombinant MMP16 (Table 2). This illustrates the possibility of KLK14 to disrupt the prodomain association with the catalytic zinc in other proMMPs in vivo, providing a universal activation mechanism for the entire proMMP family by KLK14. Nonetheless, recombinant proMMP2 also contains the corresponding ^97^MRKPR^101^C sequence; yet no KLK14-mediated activation was observed, indicating that accessibility to this sequence may be selective and depend on protein conformation (Figure 2A).

The proteolytic network interactions between the KLKs and MMPs are starting to emerge. In vitro analysis studies illustrate that MMPs can modulate KLK activity, such as MMP20 activating proKLK1, 4, and 6, as well as MMP3 activating proKLK4 [33,46]. Furthermore, major MMPs involved in tumor progression (proMMP1, 2, and 9) have been shown to be activated by KLK1, which may lead to enhanced tissue homeostasis or dysregulation [47]. Cell surface-located, membrane type MMPs have been identified as important players in bone remodeling, as well as in wound healing, growth factor signaling, and in immune functions [48,49,50], while KLKs are implicated in immune functions and TGFβ activation [51]. Interestingly, the presence of both MT-MMPs and KLKs was identified in many types of cancer [50,52]. A body of research indicates MMP14 as the main membrane type MMP essential for cancer cell escape and hence, tumor progression [53,54], which is partially attributed to proMMP2 activation, but also for the ability of MMP14 to directly degrade type I collagen and to promote cellular invasion in 3D collagen matrices [55]. Similarly, MMP15 and MMP16 were shown to promote invasiveness of tumor cells in 3D fibrin matrices [10,56], while, in particular, MMP16 is highly expressed in aggressive melanoma [10]. MMP17 was implicated as a protease critical for breast cancer metastasis in animal models [57]. KLK14 was similarly implicated in many human tumors. Most notably, KLK14 levels were found to predict poor outcomes in prostate cancer patients [58], and elevated levels of both transcript and protein were found in malignant breast cancer tissues [59,60]. In correlation, increased levels of MMP14 were detected in prostate cancer cells [61,62], and active MMP2 was found to be inversely correlated with the disease-free survival time in prostate cancer patients [61]. Furthermore, elevated levels of MMP14 were associated with poor prognosis and invasiveness in breast cancer [63,64]. Recently, the involvement of MMP14 was also indicated in epithelial-to-mesenchymal transition in squamous cell carcinoma [65,66] and prostate cancer alike [67].

It is therefore exciting to speculate, that the herein described potential of KLK14 to activate membrane-type MMPs may provide a novel mechanism facilitating prostate and breast tumorigenesis, initial cancer cell invasion, and subsequent tumor progression at the sites of metastases formation. In addition, future studies include profiling more KLKs utilizing the CleavEx analysis to better understand the proteolytic network interaction between KLKs and proMMPs.

## 4. Materials and Methods

### 4.1. Cloning of HmuY-Based CleavEx Fusion Proteins

The CleavEx fusion proteins were constructed based on a proteolysis-resistant *Porphyromonas gingivalis* HmuY protein (accession number ABL74281.1) as a carrier via PCR cloning. Firstly, the HmuY gene was amplified using primers forward: 5′–atatgcggccgcagacgagccgaaccaaccctcca–3′ and reverse: 5′–atactcgagttatttaacggggtatgtataagcgaaagtga–3′ from whole-genomic DNA isolated from *P. gingivalis* strain W83. PCR was conducted for 35 cycles with initial denaturation at 98 °C, followed by 40s annealing at 68 °C and 30 s extension at 72 °C, using Phusion DNA polymerase (Thermo Fisher Scientific, Waltham, MA, USA) and T100 Thermal Cycler (Bio-Rad, Hercules, CA, USA). The HmuY PCR product was further amplified in three consecutive PCR reactions with primers specific to the 5′ HmuY fragment and a 3′ specific primer introducing additional nucleotides dependent on the designed sequence (Table 3) at the same conditions. All sequences were designed based of the accession number from the Uniprot database (www.uniprot.org): MMP1 (P03956), MMP2 (P08253), MMP3 (P08254), MMP7 (P09237), MMP8 (P22894), MMP9 (P14780), MMP10 (P09238), MMP11 (P24347), MMP12 (P39900), MMP13 (P45452), MMP14 (P50281), MMP15 (P51511), MMP16 (P51512), MMP17 (Q9ULZ9), MMP19 (Q99542), MMP20 (O60882), MMP21 (Q8N119), MMP23 (O75900), MMP24 (Q9Y5R2), MMP25 (Q9NPA2), MMP26 (Q9NRE1), MMP27 (Q9H306), and MMP28 (Q9H239). Lastly, the final PCR reaction was ligated into a modified pETDuet plasmid, according to the manufacturers protocol, with potential tryptic cleavage sites removed from the MCS using QuickChange (Agilent Technologies, Santa Clara, CA, USA). An alternative method was also used for the fusion protein-encoding sequences by using Phusion Site-Directed Mutagenesis (Thermo Fisher Scientific, Waltham, MA, USA) via sequence exchange from a previously prepared CleavEx construct (Table 4). The final product was transformed into competent *E. coli* T10 cells and then purified and sequenced. All CleavEx_proMMP_ DNA sequences were identified to be as intended.

### 4.2. Expression and Purification of CleavEx Fusion Proteins

The designed fusion proteins covering proMMP activation sequences were expressed using an *E. coli* BL21 expression system. Following the 0.5 mM IPTG induction at OD_600nm_ = 0.5–0.6, the bacterial culture protein production was facilitated for 3 h at 37 °C, with shaking. Then, the bacteria were spun down, and the pellet was suspended in buffer A (10 mM sodium phosphate, 500 mM NaCl, and 5 mM imidazole, pH 7.4) and sonicated (15 min at 16 °C, pulse 6s, amplitude 70%). Supernatant of the soluble proteins was then loaded onto the HisTrap™ Excel (GE Healthcare, Chicago, IL, USA) column in buffer A and eluted with a linear gradient of 0–100% of 1 M imidazole in buffer A in 20 column volumes (CV). Protein containing fractions were pooled together and exchanged into 50 mM Tris pH 7.5 and then purified by ion exchange chromatography using a MonoQ 4.6/100 PE column (GE Healthcare, Chicago, IL, USA) with a linear gradient of 0–100% 50 mM Tris pH 7.5, 1 M NaCl in 15 CV. Purity of all the products was verified by SDS-PAGE.

### 4.3. Expression and Production of KLK14

The gene encoding human proKLK14 was custom-synthesized by Life Technologies (Carlsbad, CA, USA) with a codon usage optimized for *Leishmania tarentolae* and cloned into the pLEXSY_I-blecherry3 plasmid (Cat. No. EGE-243, JenaBioscience, Jena, Germany) using NotI and XbaI restriction sites. All preparations for transfection, selection, and expression in host strain T7-TR of *L. tarentolae* were performed according to the JenaBioscience protocol for inducible expression of recombinant proteins secreted to the medium. Expression of proKLK14 was induced with 15 µg/mL of tetracycline (BioShop, Burlington, Canada) and was carried out for 3 days. Next, the media was spun down (20 min at 3000 rcf), and the supernatant was precipitated with 80% ammonium sulfate for 1 h at 4 °C and then centrifuged (30 min at 15,000 rcf). The pellet was suspended in 10 mM sodium phosphate, 500 mM NaCl, and 5 mM imidazole, pH 7.4 in the presence of 5 mM benzamidine and dialyzed overnight at 4°C in the same buffer. The following day, the solution was loaded onto HisTrap™ Excel (GE Healthcare) as described above (Section 4.2). Obtained fractions were analyzed by SDS PAGE and fractions containing proKLK14 were concentrated with Vivaspin® 2 (Sartorius, Göttingen, Germany) and further purified on Superdex s75 pg (GE Healthcare, Chicago, IL, USA) in 20 mM Tris pH 7.5, 0.5 M NaCl. After purification and self-activation overnight at 4 °C, KLK14 was active-site titrated as described in Kantyka et al. [68].

### 4.4. Screening the CleavEx_proMMP_ Fusion Proteins with KLK14 and N-Terminal Sequencing of KLK14-Released Fragments

CleavEx proteins were incubated at a 1:1000 and 1:200 KLK14:CleavEx_proMMP_ molar ratio, corresponding to 50 and 250 nM KLK14, respectively, in 50 mM Tris pH 7.5. In addition, lower KLK14 concentrations (10, 25, and 50 nM) were tested on CleavEx proMMP11, 14–17, 24, 25, and 28. Samples were incubated at 37 °C for 1 h, after which the reactions were immediately stopped by the addition of 50 mM DTT-supplemented SDS sample buffer (1:1) and boiled. The obtained samples were resolved using 10% Tricine SDS-PAGE. The proteins were electrotransferred onto a PVDF membrane (Amersham™ Hybond™, GE Healthcare) in 25 mM Tris, 190 mM glycine, and 20% methanol at 100 V for 1 h in 4 °C. The membranes were blocked with 5% skim milk in TTBS (50 mM Tris-HCl, 500 mM NaCl, 0.05% Tween-20, pH 7.5) and incubated with an anti-HisTag-HRP antibody (catalog no. A7058, Sigma-Aldrich, St. Louis, MO, USA) diluted 1:20 000 in TTBS. The Western blots were developed with Pierce® ECL Western blotting substrate (Thermo Fisher Scientific, Waltham, MA, USA) using Medical X-Ray-Film Blue (Agfa HealthCare, Mortsel, Belgium).

Furthermore, each hydrolyzed CleavEx_proMMP_ (1 µg) was incubated with 250 nM KLK14 for 1 h at 37 °C, the reactions were stopped, and the products were resolved on SDS-PAGE and electrotransferred onto a PVDF membrane [69]. The membranes were stained with Coomassie Brilliant Blue R-250 (BioShop, Burlington, Canada) and the bands of interest were sequenced via automated Edman degradation using a PPSQ/31B protein sequencer (Shimadzu Biotech, Kyoto, Japan) equipped with an LC-20AT HPLC, CTO-20A column heater and SPD20A UV detector (Shimadzu Biotech) for on-line PTH analysis. Data was recorded using proprietary software (Shimadzu Biotech), and the sequence was determined by visual inspection of the UV 269 nm chromatograms.

### 4.5. SDS-PAGE Analysis of KLK14-Mediated Recombinant proMMP Processing

A total of 0.5 µg native proMMP2 (catalog no. 902-MP-010, R&D Systems, Abingdon, United Kingdom), 0.5 µg proMMP14 (catalog no. 918-MP-010, R&D Systems), 1 µg proMMP15 (catalog no. 916-MP-010, R&D Systems), 0.5 µg proMMP16 (catalog no. 1785-MP-010, R&D Systems), and 1 µg proMMP17 (catalog no. 7796-MP-010, R&D Systems) were separately incubated in 10 µL with a range of KLK14 concentrations (25–250 nM, with molar ratios from around 1:65 to 1:10 KLK14:MMP) in the presence of 5 µM batimastat (Sigma-Aldrich, St. Louis, MO, USA) for 1 h at 37 °C in PBS. As a positive control, a total of 1 µg native proMMP2 and 0.5 µg proMMP14 were separately incubated in 10 µL with a range of furin (catalog no. 1503-SE-010, R&D Systems) concentrations (0–250 nM, molar ratios from around 1:65 to 1:6). The reactions were stopped with the addition of 50 mM DTT-supplemented SDS sample buffer (1:1, *v:v*) and boiled. The samples were resolved using SDS-PAGE as described above and then visualized with Coomassie Brilliant Blue G-250 (Bioshop, Burlington, Canada) staining. Additionally, 50 nM KLK14 (proMMP14) or 100 nM KLK14 (proMMP15, proMMP16, and proMMP17) was incubated with each respective proMMP (0.5 µg proMMP14 and 16, 1 µg proMMP15 and 17) in the presence of 5 µM batimastat for specified periods of time (0–180 min). The final molar ratio for KLK14:MMP14 was 1:32, KLK14:MMP15 was 1:16, KLK14:MMP16 was 1:16, and KLK14:MMP17 was 1:18. The reaction in each sample was stopped as above and SDS-PAGE separation was visualized using Coomassie Brilliant Blue G-250.

### 4.6. N-Terminal Sequencing of KLK14 Processed Recombinant proMMPs

Each native proMMP (2 µg) was incubated with 50 nM (proMMP14) or 250 nM (proMMP15, proMMP16, proMMP17) KLK14 for 1 h at 37 °C and resolved by SDS-PAGE as described above. Proteins were then electrotransferred onto a PVDF membrane in 10 mM CAPS, 10% methanol, pH 11 using the Trans-blot semi-dry transfer cell (Bio-Rad, Hercules, CA, USA) at 15V for 30 min. The membrane was stained with Coomassie Brilliant Blue R-250 (BioShop, Burlington, Canada), and bands of interest were sequenced via Edman degradation as described above.

### 4.7. Zymogram Analysis of Recombinant proMMP by KLK14

Each native proMMP (0.5 µg) was incubated with 50 and 100 nM KLK14 (proMMP14) or 100 and 250 nM KLK14 (proMMP15, 16, and 17) for 1 h at 37 °C. After incubation, KLK14 was inhibited with 10 µM biotin-Tyr-Gly-Pro-Arg-CMK, a specific KLK14 inhibitor, and 1 µM serine protease inhibitor Kazal-type 6 (SPINK6) [70] for 15 min at 37 °C. Next, non-reducing sample buffer (1:1) was added, and samples were incubated for 30 min at 37 °C. Then, samples were separated using a Tricine-SDS gel containing 0.1% gelatin at 4 °C and 40 mA. Subsequently, the gels were washed three times with 2.5% Triton X-100, followed by overnight incubation in assay buffer with the presence of the KLK14 inhibitors at 37 °C. The assay buffer used for each proMMP was 50 mM Tris, 10 mM CaCl_2_, 150 mM NaCl, pH 7.5 (proMMP2), 50 mM Tris, 3 mM CaCl_2_, 1 μM ZnCl_2_, pH 8.5 (proMMP14 and 16), 50 mM Tris, 500 mM NaCl, 5 mM CaCl_2_, 1 μM ZnCl_2_, pH 8 (proMMP15) and 50 mM Tris, 10 mM CaCl_2_, pH 7.5 (proMMP17). The next day, the zymogram was fixed by using 30% methanol with 10% acetic acid for 2 min and stained with 0.1% amido black in 10% acetic acid for 2 h at room temperature, followed by destaining with 10% acetic acid.

### 4.8. Functional Activation of Recombinant proMMP14 Using A Synthetic Substrate

ProMMP14 (10 nM) was separately incubated with KLK14 (3 nM) and furin (3 nM) in the presence of the fluorogenic substrate Mca-KPLGL-Dpa-AR-NH_2_ (catalog no. ES010, R&D Systems, Abingdon, United Kingdom). The final substrate concentration was 10 µM in 50 mM Tris, 3 mM CaCl2, 1 µM ZnCl2, pH 8.5. Hydrolysis was recorded for 180 min at 37 °C (λ_ex_320 nm; λe_m_405 nm) using a microplate fluorescence reader (Molecular Devices Spectra Max GEMINI EM, Molecular Devices, San Jose, CA, USA). The measurement was performed in triplicate, and the initial velocities were determined via the built-in linear regression algorithm. The velocities were plotted in triplicates as mean ± SD using GraphPad Prism (GraphPad Software, La Jolla, CA, USA) using time points 0, 5, 15, 30, 60, 90, 120, 150, and 180 min.

### 4.9. Cell Surface Processing of proMMP14 (MT1-MMP) by KLK14

*Timp* 2-/- mouse embryonic fibroblast (MEF) cells stably expressing FLAG-tagged MT1-MMP transfected with the pGW1GH/hMT1-MMP expression vector [71] were seeded in a 12-well plate (250,000 cells per well) and cultured in 1.5 mL selection medium DMEM (Gibco, Waltham, MA, USA) with 10% FBS (Gibco), 25 µg/mL mycophenolic acid (Sigma-Aldrich, St. Louis, MO, USA), 250 µg/mL xanthine (Sigma-Aldrich), and 1X HT supplement (Life Technologies, Carlsbad, CA, USA) for 2 days at 37 °C, 5% CO_2_ to 80% confluency. The selection media was aspirated, and cells were gently washed with PBS and then treated with 250 µL DMEM containing either 50 nM KLK14, 50 nM furin and each enzyme preincubated (15 min at 37 °C) with its specific inhibitor: either 250 nM SPINK6 [70] or 1 µM decanoyl-RVKR-CMK (Bachem, Bubendorf, Switzerland), respectively, and further incubated for 30 min at 37 °C, 5% CO_2_. In addition, separate cell populations were incubated with increasing concentrations of KLK14 (50, 100, 250, and 500 nM) and compared to the appropriate control samples, which included: (i) untreated cells; (ii) cells treated with SPINK6 and (iii) dec-RVKR-CMK inhibitors; (iv) 50 nM furin; (v) 50 nM furin with 1 µM dec-RVKR-CMK; and (vi) 500 nM KLK14 in the presence of 1 µM SPINK6.

The supernatant was aspirated and the cells were rinsed with cold PBS three times and then treated with 0.1 mg/mL EZ-link™ Sulfo-NHS-LC-Biotin (Thermo Fisher Scientific, Waltham, MA, USA) for 1 h. Next, the cells were washed three times with PBS containing 100 mM glycine and then lysed with 500 µL RIPA buffer (10 mM Tris pH 7.5, 150 mM NaCl, 1% NP-40, 0.1% SDS, 1% DOC, 2 mM EDTA) containing 25X cOmplete™ EDTA-free Protease Inhibitor Cocktail (Roche, Mannheim, Germany) and 5 mM EDTA. Cells were detached and spun down 16,000 rcf for 15 min at 4 °C. The protein concentration of the lysate was determined using the Pierce™ BCA Protein Assay Kit (Thermo Fisher Scientific, Waltham, MA, USA) according to the manufacturer’s instructions. A total of 500 µg was loaded onto PureProteome streptavidin magnetic beads (Merck Millipore, Burlington, MA, USA) and incubated overnight at 4 °C. The beads were washed twice in RIPA buffer followed by a twice wash in PBS and then suspended in 50 mM Tris pH 7.5 and 6x reducing sample buffer to a final 3× reducing sample buffer (*v*/*v*) in a final 40 µL volume. Samples were boiled at 95 °C for 5 min, resolved on SDS-PAGE, and electrotransferred onto a PVDF membrane [69].

The membrane was subsequently blocked with 5% skim milk in TTBS (50 mM Tris-HCl, 500 mM NaCl, 0.05% Tween-20, pH 7.5) for 2 h at 37 °C. Next, primary antibody rabbit-anti-MMP14 (catalog no. MA5-32076, Thermo Fisher Scientific, Waltham, MA, USA) in 5% milk was added at 1:1000 overnight at 4 °C. The following day, the membrane was washed four times with TTBS, and secondary antibody goat-anti rabbit-HRP (catalog no. A7058, Sigma-Aldrich, St. Louis, MO, USA) was added at 1:60,000 in 5% milk for 2 h at RT. Lastly, the membrane was rinsed four times with TTBS and developed with the SuperSignal® West Femto Maximum Sensitivity Substrate (Thermo Fisher Scientific, Waltham, MA, USA) using Medical X-Ray-Film Blue (Agfa HealthCare, Mortsel, Belgium).

## Figures and Tables

**Table 1:** Identification of the KLK14 hydrolysis sites within the CleavEx_proMMP_ protein. CleavEx_proMMP_ fusion proteins were separated using SDS-PAGE and electrotransferred for N-terminal sequencing. Identified sequences are represented in the bold font and the underscore denotes where the location of the expected activation cleavage P1-P1′ in the proMMP-derived sequence.

CleavEx_proMMP_	Identified Sequence
proMMP11	RQKR^27^**FVLSDEP**
proMMP14	RRKR^27^**YAIQDE**
proMMP15	RRRKR^28^**YALTGRD**
proMMP16	RRKR^27^**YALTDEP**
proMMP17	ARRRR^28^**QAPAPTDE**
proMMP21	ARSRRSPR^31^**APLDEPN**
proMMP23	RRRR^27^**YTLPDEPN**
proMMP24	RRNKR^28^**YALTGQDE**
proMMP25	RRRRR^28^**YALSGSDE**
proMMP28	RRKKR^28^**FAKQGNDE**

**Table 2:** N-terminal identification of KLK14-mediated processing of recombinant proMMPs. The KLK14 hydrolysis product sequences were analyzed by N-terminal sequencing using Edman degradation. The bold font denotes the amino acid sequences identified. The underscored residues represent changes to the native protein sequence, as reported by the manufacturer (R&D Systems, Abingdon, United Kingdom). KLK14 recognized the sequence 3-aa upstream of the native MMP17 activation site, likely because the native site was modified by the manufacturer. All residues are numbered according to the Uniprot reported sequence of the full-length proteins. Bands are labeled according to the notation explained at Figure 2.

Protease	Band	N-Terminal Sequence	Annotation
**proMMP14**	1	NH_2_-ALAS^24^**LGSAQSSSFS**	Proform
	2	RRKR^111^**YAIQGLKWQH**	Active form
**proMMP15**	1	**NH_2_-E^47^DAEVHAENWLLY**	Proform
	2	PGKR^131^**YALTGRKWNH**	Active form
	3	DLRG^298^**IQQLYGTPDGQP**	Hinge region
**proMMP16**	1	**NH_2_-A^32^TVCGTEQYF**	Proform
	2	ALAA^75^**MQQFYGINMT**	Truncated proform
	3	KKPR^100^**CGVPDQTRGS**	Truncated proform
	4	RRKR^119^**YALTGQKWQ**	Active form
**proMMP17**	1	**NH_2_-A^39^PAPAPRAEDLSL**	Proform
	2	**NH_2_-A^39^PAPAPRAEDLSL**	Proform
	3	TQAR^122^**RRPQAPAPTKW**	Active form
	4	TQAR^122^**RRPQAPAPTKW**	Active form

**Table 3:** Primers used for generating the proMMP CleavEx fusion proteins using three consecutive PCRs.

Protein Name	Primer	Primer Sequence (5′-3′)
	reverse *	ATATGCGGCCGCTTATTTAACGGGGTATGTATAAGCGA
proMMP15	forward 1	GCCCTCACCGGGAGGGACGAGCCGAACCAACCCT
proMMP15	forward 2	CGGAAGCGCTACGCCCTCACCGGGAGGGAC
proMMP15	forward 3	ATATGTCGACCGGCGTCGGAAGCGCTACGCCCTCA
proMMP17	forward 1	GCTCCAGCCCCCACCGACGAGCCGAACCAACCCT
proMMP17	forward 2	AGGAGACGCCAGGCTCCAGCCCCCACCGAC
proMMP17	forward 3	ATATGTCGACGCTCGCAGGAGACGCCAGGCTCCAG
proMMP19	forward 1	CTGTTGCTGGGCCGCGACGAGCCGAACCAACCCT
proMMP19	forward 2	ACCCTTAAATACCTGTTGCTGGGCCGCGAC
proMMP19	forward 3	ATATGTCGACCAGAAGACCCTTAAATACCTGTTGCTGG
proMMP21	forward 1	CCGCGGGCGCCGCTGGACGAGCCGAACCAACCCT
proMMP21	forward 2	TCCAGGCGCTCCCCGCGGGCGCCGCTGGAC
proMMP21	forward 3	ATATGTCGACGCCCGCTCCAGGCGCTCCCCGCGG
proMMP24	forward 1	GCCCTGACTGGACAGGACGAGCCGAACCAACCCT
proMMP24	forward 2	GAAACAAGCGCTATGCCCTGACTGGACAGGAC
proMMP24	forward 3	ATATGTCGACCGGAGAAACAAGCGCTATGCCCT
proMMP25	forward 1	CTGAGCGGCAGCGACGAGCCGAACCAACCCT
proMMP25	forward 2	CGCCGGTACGCTCTGAGCGGCAGCGAC
proMMP25	forward 3	ATATGTCGACAGGCGGCGTCGCCGGTACGCTCTGAGC
proMMP28	forward 1	GCAAAGCAAGGTAACGACGAGCCGAACCAACCCT
proMMP28	forward 2	TAAGAAACGCTTTGCAAAGCAAGGTAACGACGAG
proMMP28	forward 3	ATATGTCGACAGGCGTAAGAAACGCTTTGCAAAGCAAG

* = the same reverse primer was used for all of the first step PCR reactions with each subsequent forward 1 primer, respectively.

**Table 4:** Primers used for generating the proMMP CleavEx fusion proteins using mutagenesis.

Protein Name	Primer	Primer Sequence (5P′-3′)
	reverse *	GTCGACCTGCAGGCTCGC
proMMP1	forward	GAUGUGGCGCAGUUUGUGCUGACCGACGAGCCGAACCAACCC
proMMP2	forward	GAUGUGGCGAACUAUAACUUUUUUGACGAGCCGAACCAACCC
proMMP3	forward	GAUGUGGGUCAUUUUCGUACCUUUGACGAGCCGAACCAACCC
proMMP7	forward	GAUGUGGCGGAAUAUAGCCUGUUUGACGAGCCGAACCAACCC
proMMP8	forward	GGUGGUUUUAUGCUGACCCCGGGUGACGAGCCGAACCAACCC
proMMP9	forward	GAUCUGGGU CGUUUUCAGACCUUUGACGAGCCGAACCAACCC
proMMP10	forward	GAUGUGGGUCAUUUUAGCAGCUUUGACGAGCCGAACCAACCC
proMMP11	forward	CGUCAGAAACGUUUUGUGCUGAGCGACGAGCCGAACCAACCC
proMMP12	forward	GAAAUGCCGGGUGGUCCGGUGUGGGACGAGCCGAACCAACCC
proMMP13	forward	GAUGUGGGUGAAUAUAACGUGUUUGACGAGCCGAACCAACCC
proMMP14	forward	CGUCGUAAACGUUAUGCGAUUCAGGACGAGCCGAACCAACCC
proMMP16	forward	CGUCGUAAACGUUAUGCGCUGACCGACGAGCCGAACCAACCC
proMMP20	forward	GAUGUGGCGAACUAUCGUCUGUUUGACGAGCCGAACCAACCC
proMMP23	forward	CGUCGUCGUCGUUAUACCCUGCCGGACGAGCCGAACCAACCC
proMMP26	forward	GAUGGUAGCGAUACCAGCAUUAGCGACGAGCCGAACCAACCC
proMMP27	forward	GAUGUGGGUCAGUAUGGUUAUACCGACGAGCCGAACCAACCC

* = the same reverse primer was used for each PCR reaction, respectively.

## Supplementary Materials

The following are available online at https://www.mdpi.com/1422-0067/21/12/4383/s1, Figure S1. Effect of KLK14 on select CleavEx_proMMP_ fusion proteins. Figure S2. (A) Processing of cell surface proMMP14 by KLK14. (B) Densitometry analysis of the 56 kDa band using ImageJ, the data is represented as a log of arbitrary density units.

## Abbreviations

ADAM	A disintegrin and metalloproteinase
CleavEx	Cleavage of exposed amino acid sequences
ECM	Extracellular matrix
HmuY	Heme-binding protein
KLK	Kallikrein-related peptidase
MMP	Matrix metalloproteinase
MT-MMP	Membrane-type matrix metalloproteinase
MT1-MMP	Membrane-type 1 matrix metalloproteinase, MMP14
TMPRSS2	Transmembrane serine protease 2
