# Supplementary material for: Kallikrein-Related Peptidase 14 Activates Zymogens of Membrane Type Matrix Metalloproteinases (MT-MMPs)—A CleavEx Based Analysis
Source: Int J Mol Sci. 2020 Jun 19;21(12):4383. doi: 10.3390/ijms21124383 (PMC7352328; doi:10.3390/ijms21124383)
Supplement: Supplementary file 1 [file ijms-21-04383-s001.pdf]

International Journal of *Molecular Sciences*

Supplementary Figures

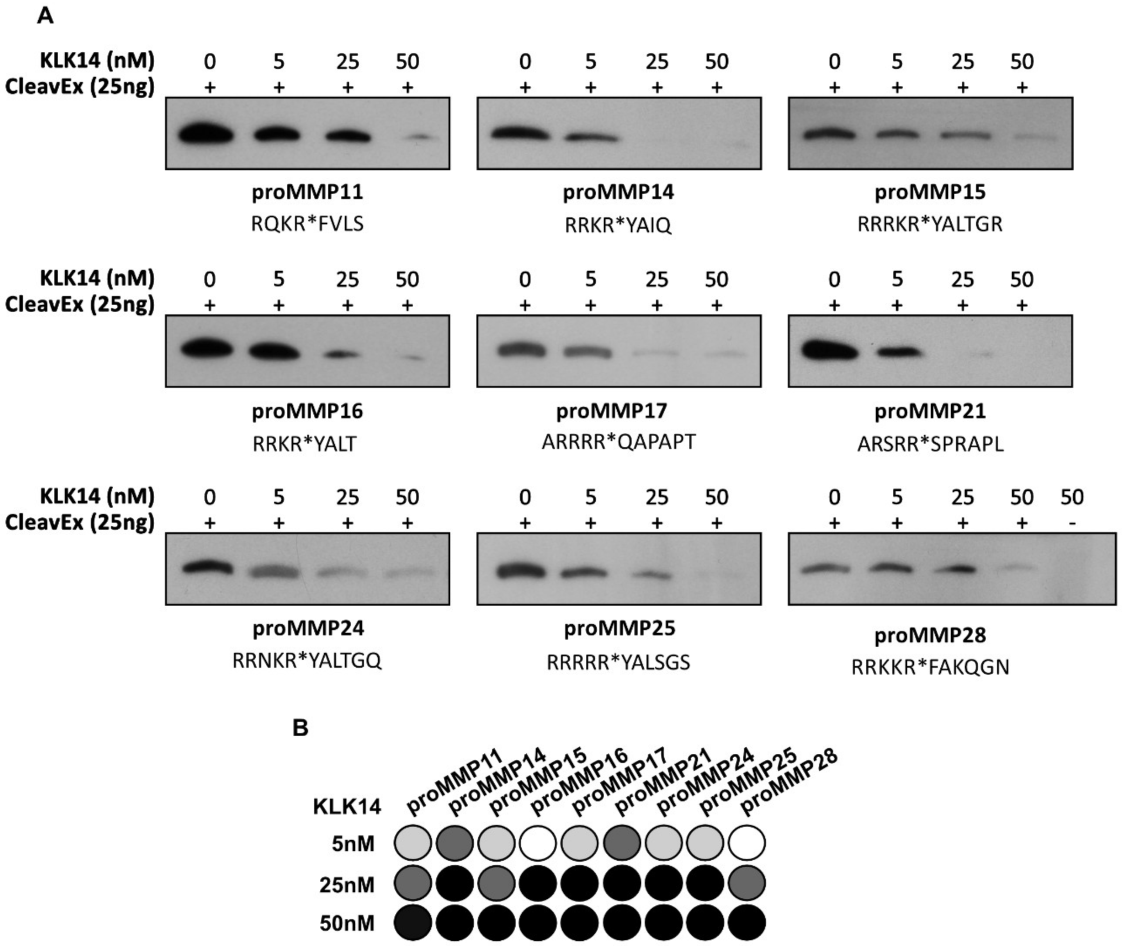

Figure S1. Effect of KLK14 on select CleavEx$_{proMMP}$ fusion proteins. (**A**) Western blot analysis of each 25 ng CleavEx$_{proMMP}$ protein incubated with 5, 25 and 50 nM KLK14 after 1 h at 37 °C. Each fusion protein with its respective activation sequence is listed with the native site of hydrolysis indicated by an asterisk. (**B**) Schematic representation of the CleavEx$_{proMMP}$ fusion proteins from the Western blots in panel A. Scoring was performed by densitometry analysis using ImageJ. The shading is based on the quartile of change: 100–75% of control sample intensity is presented as white (no degradation); 75–50% as light grey; 50–25% as dark grey, and 25% and lower as black. KLK = kallikrein-related peptidase; CleavEx = **Cleav**age of **ex**posed amino acid sequences; MMP = matrix metalloproteinase.

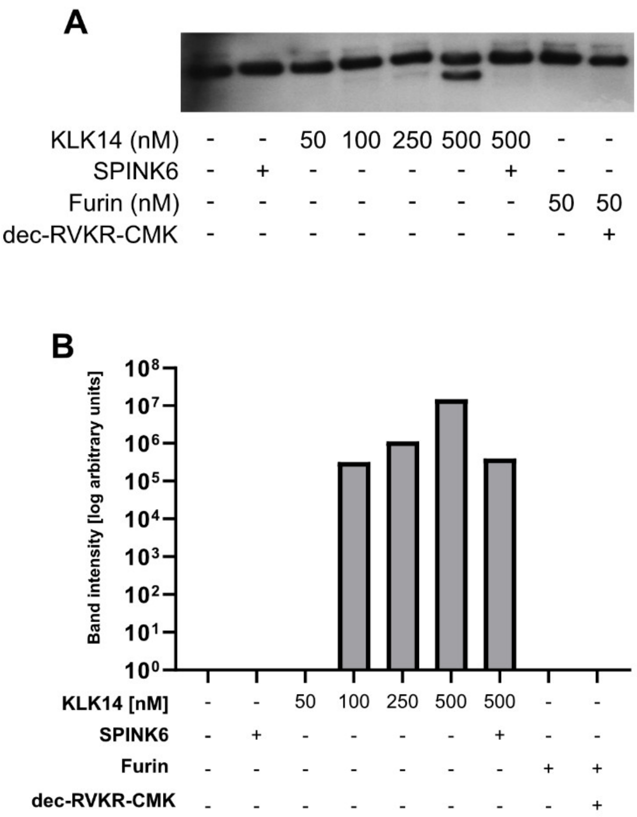

Figure S2. (**A**) Processing of cell surface proMMP14 by KLK14. Murine fibroblasts stably expressing human MMP14 (MT1-MMP) were treated with 50, 100, 250, and 500 nM KLK14 and 50 nM furin. Selective inhibitors serine protease inhibitor Kazal-type 6 (SPINK6) (KLK14) and dec-RVKR-CMK (furin) were used to inhibit KLK and furin in the control samples. Cell surface proteins were then biotinylated and streptavidin bead immunoprecipitates were subjected to immunoblotting using an anti-MMP14 antibody. Each sample contained the 63 kDa proMMP14 form, whereas an increase in the active 58 kDa MMP14 form was observed after KLK14 incubation. Additionally, a lower molecular weight MMP14 form at 56 kDa was detected only in the KLK14 treated sample. (**B**) Densitometry analysis of the 56 kDa band using ImageJ; the data is represented as a log of arbitrary density units. KLK = kallikrein-related peptidase; MMP = matrix metalloproteinase.

© 2020 by the authors. Submitted for possible open access publication under the terms and conditions of the Creative Commons Attribution (CC BY) license (http://creativecommons.org/licenses/by/4.0/).
